# Mental health interventions by lay counsellors: a systematic review and meta-analysis

**DOI:** 10.2471/BLT.20.269050

**Published:** 2021-04-29

**Authors:** Suzanne M Connolly, Michelle Vanchu-Orosco, Jan Warner, Pegah A Seidi, Jenny Edwards, Elisabeth Boath, AC Irgens

**Affiliations:** a 70 Payne Place, Suite 6, Sedona, AZ 86446, United States of America (USA).; b Greater Victoria Coalition to End Homelessness, Victoria, Canada.; c Department of Social Work Services, Veterans Affairs Medical Center, Cleveland, USA.; d Research Center, University of Garmian, Kalar, Iraq.; e School of Leadership Studies, Fielding Graduate University, Santa Barbara, USA.; f Department of Social Work and Social Welfare, Staffordshire University, Stoke-on-Trent, England.; g Department of Psychiatry, Sørlandet Hospital, Arendal, Norway.

## Abstract

**Objective:**

To investigate the effectiveness of community-based mental health interventions by professionally trained, lay counsellors in low- and middle-income countries.

**Methods:**

We searched PubMed®, Cochrane Central Register of Controlled Trials, PROSPERO and EBSCO databases and professional section publications of the United States National Center for PTSD for randomized controlled trials of mental health interventions by professionally trained, lay counsellors in low- and middle-income countries published between 2000 and 2019. Studies of interventions by professional mental health workers, medical professionals or community health workers were excluded because there are shortages of these personnel in the study countries. Additional data were obtained from study authors. The primary outcomes were measures of post-traumatic stress disorder, depression, anxiety and alcohol use. To estimate effect size, we used a random-effects meta-analysis model.

**Findings:**

We identified 1072 studies, of which 19 (involving 20 trials and 5612 participants in total) met the inclusion criteria. Hedges' *g* for the aggregate effect size of the interventions by professionally trained, lay counsellors compared with mostly either no intervention or usual care was −0.616 (95% confidence interval: −0.866 to −0.366). This result indicates a significant, medium-sized effect. There was no evidence of publication bias or any other form of bias across the studies and there were no extreme outliers among the study results.

**Conclusion:**

The use of professionally trained, lay counsellors to provide mental health interventions in low- and middle-income countries was associated with significant improvements in mental health symptoms across a range of settings.

## Résumé

Objectif Évaluer l'efficacité des interventions de santé mentale gérées au sein de la sphère communautaire par des conseillers non professionnels spécialement formés dans les pays à faibles et moyens revenus.

Méthodes Nous avons mené nos recherches dans les bases de données de PubMed®, du Registre central Cochrane des essais contrôlés, de PROSPERO et d'EBSCO, ainsi que parmi les publications de la section professionnelle du Centre national américain du TSPT, afin d'en extraire des essais randomisés contrôlés portant sur des interventions de santé mentale gérées par des conseillers non professionnels spécialement formés dans les pays à faibles et moyens revenus, publiés entre 2000 et 2019. Les études consacrées aux interventions effectuées par des professionnels de la santé mentale, des membres du corps médical ou des agents de santé communautaires ont été écartées, car les pays observés manquent de personnel dans ce domaine. D'autres données ont été prélevées auprès des auteurs d'études. Les premiers résultats étaient des mesures relatives aux troubles de stress post-traumatique, à la dépression, à l'anxiété et à la consommation d'alcool. Nous avons employé un modèle de méta-analyse à effets aléatoires pour évaluer les retombées.

Résultats Nous avons identifié 1072 études; 19 d'entre elles (impliquant 20 essais et 5612 participants au total) correspondaient aux critères d'inclusion. Le g de Hedges pour la taille de l'effet cumulé des interventions gérées par des conseillers non professionnels spécialement formés, comparé la plupart du temps avec l'absence d'intervention ou les soins habituels, s'élevait à −0,616 (intervalle de confiance de 95%: −0,866 à −0,366). Ce résultat témoigne d'un effet non négligeable, de taille moyenne. Il n'existait aucune preuve indiquant un biais de publication ou toute autre forme de biais dans les études, et les résultats ne laissaient transparaître aucune valeur extrême atypique.

Conclusion Le recours à des conseillers non professionnels spécialement formés pour effectuer des interventions de santé mentale dans les pays à faibles et moyens revenus a entraîné une amélioration significative des symptômes dans de nombreuses situations.

## Resumen

**Objetivo:**

Investigar la eficacia de las intervenciones de salud mental basadas en la comunidad y llevadas a cabo por asesores no especializados con formación profesional en países con ingresos bajos y medios.

**Métodos:**

Se realizaron búsquedas en las bases de datos PubMed®, el Registro Cochrane Central de Ensayos Controlados (Cochrane Central Register of Controlled Trials), PROSPERO y EBSCO y en las publicaciones de la sección profesional del National Center para TEPT de los Estados Unidos para obtener ensayos controlados aleatorios de intervenciones de salud mental realizadas por consejeros legos con formación profesional en países con ingresos bajos y medios, publicados entre 2000 y 2019. Se excluyeron los estudios de intervenciones realizadas por trabajadores profesionales de la salud mental, profesionales médicos o trabajadores de la salud de la comunidad porque hay escasez de este personal en los países de estudio. Se obtuvieron datos adicionales de los autores de los estudios. Los resultados primarios fueron medidas de trastorno de estrés postraumático, depresión, ansiedad y consumo de alcohol. Para estimar el tamaño del efecto, se utilizó un modelo de metanálisis de efectos aleatorios.

**Resultados:**

Se identificaron 1072 estudios, de los cuales 19 (con 20 ensayos y 5612 participantes en total) cumplieron los criterios de inclusión. El *g* de Hedges para el tamaño del efecto agregado de las intervenciones realizadas por asesores no especializados con formación profesional en comparación con la mayoría de las veces de ninguna intervención o la atención habitual fue de -0,616 (intervalo de confianza del 95%: -0,866 a -0,366). Este resultado indica un efecto significativo de tamaño medio. No hubo evidencia de sesgo de publicación o cualquier otra forma de sesgo en los estudios, así como tampoco hubo valores extremos entre los resultados de los estudios.

**Conclusión:**

El uso de asesores no especializados con formación profesional para realizar intervenciones de salud mental en países con ingresos bajos y medios se asoció con mejoras significativas en los síntomas de salud mental en una serie de entornos.

## ملخص

الغرض التحقيق في مدى فعالية تدخلات الصحة النفسية المعتمدة على المجتمع، بواسطة مستشارين غير متخصصين ومدربين تدريباً مهنياً في الدول منخفضة الدخل ومتوسطة الدخل.

الطريقة بحثنا في ®PubMed، وسجل كوكرين المركزي للتجارب ذات الشواهد، وقواعد بيانات PROSPERO وEBSCO، ومنشورات القسم المهني للمركز الوطني للولايات المتحدة لـ PTSD، من أجل التجارب العشوائية ذات الشواهد لتدخلات الصحة العقلية بواسطة مستشارين محترفين غير متخصصين ومدربين تدريباً مهنياً من الدول منخفضة الدخل ومتوسطة الدخل، والمنشورة بين عامي 2000 و2019. تم استبعاد دراسات التدخلات التي تمت بواسطة العاملين في مجال الصحة العقلية المهنية، أو المهنيين الطبيين، أو العاملين في القطاع الصحي المجتمعي، وذلك لوجود نقص في هؤلاء الأفراد في الدول التي تمت بها الدراسة. تم الحصول على بيانات إضافية من مؤلفي الدراسة. كانت النتائج الأولية بمثابة مقاييس لاضطراب إجهاد ما بعد الصدمة، والاكتئاب، والقلق، وتعاطي الكحول. ولتقدير حجم التأثير، قمنا باستخدام نموذج التحليل التلوي للتأثيرات العشوائية.

النتائج قمنا بتحديد 1072 دراسة، استوفت 19 منها معايير التضمين (حيث شملت 20 تجربة و5612 مشاركًا بشكل إجمالي). كانت التحوطات ( *g *) لحجم التأثير الكلي للتدخلات بواسطة المستشارين غير المتخصصين والمدربين تدريباً مهنياً، مقارنة مع عدم التدخل في الغالب، أو الرعاية المعتادة، هي -0.616 (فاصل الثقة 95%: -0.866 إلى -0.366). تشير هذه النتيجة إلى تأثير ملموس متوسط ​​الحجم. لم يكن هناك دليل على تحيز النشر أو أي شكل آخر من أشكال التحيز عبر الدراسات، ولم تكن هناك قيم شديدة التطرف بين نتائج الدراسة.

الاستنتاج إن الاستعانة بالمستشارين غير المتخصصين والمدربين تدريباً مهنياً لتقديم تدخلات الصحة النفسية في الدول منخفضة الدخل ومتوسطة الدخل، كان مرتبطًا بتحسينات ملموسة في أعراض الصحة النفسية عبر مجموعة من الأوضاع.

## 摘要

**目的:**

旨在调查低收入和中等收入国家由受过专业培训的非专业咨询师开展的以社区为基础的心理健康干预措施的有效性。

**方法:**

我们搜索了 PubMed®、Cochrane 对照试验中心注册资料库、PROSPERO 和 EBSCO 数据库以及美国国家创伤后应激障碍中心发表的专业出版物，以查看在 2000 年至 2019 年期间发表的低收入和中等收入国家经过专业培训的非专业咨询师开展的心理健康干预措施的随机对照试验。因为我们所研究的国家中此类专业资源紧缺，所以调查中未纳入针对专业心理健康工作者、专业医疗人员或社区卫生工作者所开展干预措施的研究。其他的补充数据来自研究作者。主要指标是对创伤后应激障碍、抑郁、焦虑和饮酒情况进行衡量。我们采用随机效应元分析模型来估算效应大小。

**结果:**

我们确定了 1072 项研究，其中 19 项（包括 20 项试验和共计 5612 名参与者）符合纳入标准。受过专业培训的非专业咨询师开展的干预措施与大多数没有干预措施或常规护理相比，其总体效应大小 Hedges' *g* 值为- 0.616（95% 置信区间：-0.866 至-0.366）。这一结果表明存在显著的中等效应。没有证据表明研究中存在发表偏倚或任何其他形式的偏倚，而且研究结果中也没有极端的离群值。

**结论:**

在低收入和中等收入国家，使用受过专业培训的非专业咨询师提供心理健康干预措施与各种环境下心理健康症状的显著改善存在联系。

## Резюме

**Цель:**

Изучить эффективность мероприятий по охране психического здоровья на уровне местных сообществ, проводимых в странах с низким и средним уровнем доходов непрофессиональными консультантами, получившими специализированную подготовку.

**Методы:**

Авторы провели поиск по базам данных PubMed®, Кохрановского центрального реестра контролируемых исследований, PROSPERO и EBSCO, а также по публикациям профессиональных секций Национального центра США по вопросам ПТСР; предметом поиска были опубликованные в период с 2000 по 2019 год рандомизированные контролируемые исследования вмешательств в области психического здоровья, проводимые в странах с низким и средним уровнем доходов непрофессиональными консультантами, получившими специализированную подготовку. Исследования вмешательств, проводимых силами специалистов в области охраны психического здоровья, медицинских работников или медико-санитарных работников, были исключены из-за нехватки этих кадров в исследуемых странах. Дополнительные данные были получены от авторов исследований. Первичными исходами были измерения посттравматического стрессового расстройства, депрессии, беспокойства и употребление алкоголя. Для оценки размера эффекта авторы использовали модель метаанализа случайных эффектов.

**Результаты:**

Было выявлено 1072 исследования, 19 из которых (с участием 20 исследований и 5612 участников в целом) соответствовали критериям включения. Значение параметра g по Хеджесу для совокупного размера эффекта вмешательств непрофессиональных консультантов, прошедших специализированную подготовку, по сравнению с отсутствием вмешательства или обычным лечением составляло –0,616 (95%-й ДИ: от −0,866 до −0,366). Этот результат указывает на значимый эффект средней величины. Не было доказательств публикационного смещения или какой-либо другой формы предвзятости в исследованиях, и не было никаких резко отклоняющихся значений среди результатов исследования.

**Вывод:**

Привлечение прошедших специальную подготовку непрофессиональных консультантов для проведения мероприятий по охране психического здоровья в странах с низким и средним уровнем доходов привело к значительному улучшению симптомов психического здоровья в различных условиях.

## Introduction

A recent reappraisal of the global burden of mental illness using a broad definition of mental illness as a disease concluded that it accounted for a greater percentage of the global burden of disease, in terms of years lost to disability, than any other disease category.^1^ Moreover, according to the World Health Organization (WHO), in 2011 between 76% and 85% of people with mental illnesses in low- and middle-income countries went untreated.^2^ This gap was partly due to a shortage of mental health professionals and to resources being concentrated in large, centrally located institutions rather than in community settings.^3^

In many places in the world, there may be only one psychiatrist for every 500 000 people and most professional mental health resources are taken up by patients with severe mental illnesses.^4^ This problem has been exacerbated by the coronavirus disease 2019 pandemic, which has further challenged people’s psychological and physical resilience.^5^^,^^6^ The use of community health workers has been examined as a partial solution to the shortage of mental health workers. However, in many settings, there are few community health workers, they are overburdened and little research has been performed into their cost–effectiveness in providing mental health interventions.^7^

In 2016, WHO predicted a shortage of 18 million health workers (including medical doctors, nurses and community health workers) in low- and middle-income countries by 2030.^8^ One proposed solution is to use professionally trained, lay counsellors to provide mental health interventions. In 2003, WHO’s Department of Mental Health and Substance Dependence held a meeting to address the gap in mental health provision in low- and middle-income countries after large-scale disasters and conflicts.^9^ Meeting participants highlighted the importance of maximizing community resources. Subsequently, guidelines for emergency relief efforts created by the Inter-Agency Standing Committee proposed the use of tiered care and the committee recommended that mental health interventions could be delivered by trained, nonprofessional, community members.^10^ People who need additional help could be referred to mental health professionals.

The Grand Challenges in Global Mental Health initiative, launched by the United States National Institutes of Health and several global organizations, met in 2010 and created a list of 40 grand challenges in response to the shortage of mental health services in low- and middle-income countries.^11^ Recommendations included the development of sustainable models of training for, and increasing the number of, ethnically diverse lay and specialist mental health service providers. In addition, the *Lancet* Commission on Global Mental Health and Sustainable Development noted in 2018 that there had recently been a shift from reliance on a single group of experts for providing mental health services towards the use of nonspecialist providers such as teachers, community health workers, law enforcement officers and people with lived experience.^12^

Yet, there have been few studies of mental health interventions facilitated by professionally trained, lay community members, particularly in situations where professional resources are scarce. Nevertheless, there is evidence suggesting that lay community workers can effectively provide mental health interventions in low-resource communities.^13^ For this review, we investigated the potential for expanding health resources rather than taxing already overburdened health workers. As we could find no universally accepted definition of the term, we defined a lay counsellor as a professionally trained member of the community who had no specific mental health training before being trained in the use of one or more mental health interventions.

The aim of our literature review was to investigate the effectiveness of community-based mental health interventions facilitated by professionally trained, lay counsellors in low- and middle-income countries at the level of the community. Involving the community as agent of change is the least-used category of community-based interventions in public health, and this strategy can promote healthier communities and strengthen the community’s capacity to address health issues.^14^

Although previous reviews of treatment for mental disorders in low- and middle-income countries have sometimes included the use of lay counsellors,^15^^–^^21^ our review examines exclusively the effectiveness of professionally trained, lay counsellors from the community in treating common mental disorders in low- and middle-income countries.

## Methods

This review is registered with PROSPERO (CRD42019118999). Our literature review included studies that involved the provision of mental health interventions by lay counsellors living in the local community in low- and middle-income countries. In eligible studies, counsellors were drawn from a broad cross section of the community and were exclusively individuals who were not employed as medical or mental health professionals, teachers or community health workers and who did not work for nongovernmental organizations or government institutions.

Study inclusion criteria were selected by two authors and a literature search found that no prior review used the same criteria. Eligible studies had evaluated the use of professionally trained, lay counsellors to facilitate mental health interventions in low- and middle-income countries in a randomized controlled trial published between 2000 and 2019. We also included preventive studies or studies not in English. We did exclude studies that used closely related interventions, such as professionally led self-help groups, media-distributed interventions and interventions involving peer support (i.e. involving individuals who derived their knowledge from personal experience rather than formal training).^22^ We also excluded studies of interventions by professional mental health workers, medical professionals or community health workers.

We searched the PubMed®, Cochrane Central Register of Controlled Trials, PROSPERO and EBSCO (EBSCO Information Services, Ipswich, United States of America, USA) databases using the search terms *lay counsellors*, *mental health interventions in low and middle income countries*, *mental health interventions after disasters*, *lay counsellors mental health*, *community member facilitated mental health*, *lay counsellor mental health Africa randomised controlled trials*, *community mental health interventions in LMICs*, *community-member facilitated mental health randomised controlled trials*, *cognitive behavioral therapy based intervention by community health workers* and *task-shifting*. In addition, we searched the professional section publications of the United States National Center for PTSD (United States Department of Veterans Affairs, Washington DC, USA) using the terms *posttraumatic stress disorder research* and *PTSD research*. We also examined reference sections of the relevant studies identified.

The titles and abstracts of studies identified by the search were examined by two authors according to preferred reporting items for systematic reviews and meta-analyses guidelines using a flow diagram for data extraction designed for this review:^23^ Details are available from the data repository.^24^ Disagreements about which studies to include were discussed until agreement was reached, sometimes in consultation with a third author. One author extracted data from the selected studies and data accuracy was cross-checked by another. Study authors were contacted, where relevant, to obtain data that were not included in the study but were necessary for the meta-analysis or for assessing the risk of bias. Where several papers reported the same data, the data were included only once. An assessment of inter-rater reliability of coding found that 98.7% of data entries were in agreement, with only 2 of 148 data points having to be changed (data repository).^24^

### Data analysis

We performed the meta-analysis using Comprehensive Meta-Analysis v. 2.2.050 (Biostat, Englewood, USA). Sample sizes and the means and standard deviations of outcome measures needed for the meta-analysis were obtained from the study publications. Effect sizes were derived from differences in the means of outcome measures at the first assessment following the intervention between individuals who received mental health interventions from lay counsellors and those in control groups. Both these effect sizes and an aggregate effect size are expressed using Hedges’ *g* coefficient. For this meta-analysis we used direct measures with negative numbers reflecting a reduction in symptoms after treatment. Effect sizes were calculated using the pooled standard deviation.^25^, categorized them as previously suggested:^26^ (i) Hedges’ *g* between 0.0 and ±0.2 indicates no effect; (ii) from > ±0.2 to < ±0.5 indicates a small effect; (iii) from > ±0.5 to < 0.8 indicates a medium effect; and (iv) ≥ ±0.8 indicates a large effect.

As the variation in outcome measures was likely to exceed the sampling error, we decided to use a random-effects meta-analysis model. With a random-effects model, there are likely to be fewer issues with type-I statistical errors (i.e. false-positive findings) and confidence intervals are generally more precise. Further, the calculated confidence intervals are less likely to overstate the degree of precision of the meta-analysis.^25^ We examined the homogeneity of the variance in, and the distribution of, effect sizes between the studies using a normal Q–Q plot (SPSS, IBM, Chicago, USA). We performed an outlier analysis to better understand the contribution of individual effect sizes to the aggregate effect size and to assess their overall impact.

We used standardized Cochrane procedures to assess the potential risk of bias over seven domains: (i) random sequence generation; (ii) concealment of group allocation; (iii) quality of blinding of participants and personnel; (iv) blinding of outcome assessments; (v) reporting of incomplete outcome data; (vi) outcome reporting; and (vii) other sources of bias.^27^ Each domain was judged as having a high, unclear or low potential risk of bias. Six authors independently assessed the risk of bias in six or seven studies each and reached a consensus with another evaluator. Disagreements were resolved by involving another author. Authors did not participate in the assessment or approval of a study if they were involved in the study or personally knew one of the study’s authors.

## Results

**Fig. 1:**
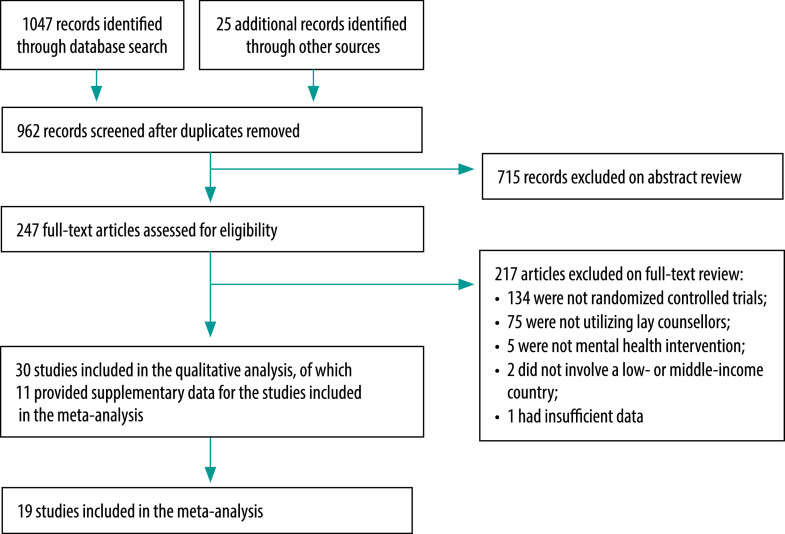
Study selection, systematic review of mental health interventions by lay counsellors in low- and middle-income countries, 2000–2019

We identified 1072 studies and, in addition, we examined the reference sections of three systematic literature reviews and meta-analyses and one other study (Fig. 1).^18^^,^^20^^,^^21^^,^^28^ After removing 110 duplicate records, the remaining 962 studies were screened by reading the abstracts or quickly reviewing the data. Subsequently, we assessed the full texts of 247 studies to determine whether they met the inclusion criteria and 217 were excluded. The characteristics of the studies excluded on the basis of abstract or full text reviews are available from the data repository.^24^ Finally, 19 studies met the inclusion criteria.^29^^–^^47^ In addition, we identified 11 supporting papers that provided additional data on studies reported in these 19 papers.^48^^–^^58^ Where more than one paper was associated with a particular study, we included the paper that contained the most information pertinent to the inclusion criteria or analyses. As one study reported two trials, the meta-analysis involved data from a total of 20 trials. One author was involved in two of the studies included and two authors personally knew an author of the study by Robson et al.;^37^ consequently, they did not participate in the assessment or approval of the studies concerned. Researchers from six studies were contacted to provide data needed for the meta-analysis that had not been included in the published articles.^29^^,^^38^^–^^40^^,^^43^^,^^44^

### Study characteristics

Of the 19 studies, 10 were conducted in Africa and nine in Asia. The primary outcomes were: (i) post-traumatic stress disorder (13 studies);^29^^–^^41^ (ii) depression (three studies);^42^^–^^44^ (iii) alcohol use (two studies);^45^^,^^46^ and (iv) anxiety and depression combined (one study).^47^ A list of the tools used to assess these outcomes is available from the data repository.^24^ The primary intervention methods facilitated by lay counsellors were: (i) cognitive behavioural therapy (six studies);^32^^,^^34^^,^^38^^–^^40^^,^^47^ (ii) individualized combinations of behavioural therapy and psychoeducation (six studies);^36^^,^^41^^,^^42^^,^^44^^–^^46^ (iii) thought field therapy (three studies);^29^^,^^30^^,^^37^ (iv) narrative exposure therapy (two studies);^31^^,^^35^ and (v) interpersonal psychotherapy (two studies).^33^^,^^43^ People in control groups: (i) were on a waiting list in 11 studies;^29^^–^^33^^,^^36^^–^^41^ (ii) received enhanced care in four studies;^43^^–^^46^ (iii) received usual care in two studies;^34^^,^^42^ and (iv) received no treatment in two studies.^35^^,^^47^

The length of training for new lay counsellors ranged from 2 days in three thought field therapy studies^29^^,^^30^^,^^37^ to 1 year in two classroom-based cognitive behavioural therapy studies (Table 1 and Table 2).^38^^,^^39^ One study involved previously trained lay counsellors who received one additional full day of training.^41^ The number of interventions facilitated by lay counsellors varied from one session (three thought field therapy studies)^29^^,^^30^^,^^37^ to 15 sessions (four classroom-based cognitive behavioural therapy studies).^32^^, ^^38^^–^^40^ In addition, 14 studies reported that tools were used to ascertain treatment fidelity and 15 studies reported that support and supervision were provided for lay counsellors during treatment. Although the lay counsellors’ roles were not specifically described in the studies, they could be readily inferred from descriptions of training, supervision and other study characteristics.

**Table 1:** Lay counsellor characteristics, systematic review of mental health interventions by lay counsellors in low- and middle-income countries, 2000–2019

Study	Lay counsellor selection	Lay counsellor training
Ali et al. (2003)^47^	(i) Women from the community were recruited by word of mouth and distribution of leaflets; and (ii) 12 women were selected on the basis of their communication skills, motivation, literacy in Urdu and freedom to move about	Eleven 3-hour training sessions on a cognitive behavioural therapy-based intervention over 4 weeks
Neuner et al. (2008)^35^	(i) Nine refugees (five women and four men; mean age: 27 years) from the community were trained as counsellors. Skills required to be accepted for the training included literacy in English and literacy in their mother tongue, as well as the ability to empathize with their clients and a strong motivation to carry out this work; and (ii) their educational level varied from primary school to university education	6 weeks of education in counselling for alcohol problems, a psychoeducational and social skills intervention, and in general counselling skills
Tol et al. (2008)^40^	(i) An unspecified number of lay counsellors, or interventionists, who had to be older than 17 years and have at least a high school education, were selected from local target communities based on a selection procedure assessing social skills through role plays; (ii) they were generally people with no formal mental health training but had some experience as volunteers in humanitarian programmes; and (iii) a study author stated in email correspondence that “the interventionists were newly hired community members, not currently employed as community health workers, etc.”	Once selected, interventionists received a 2-week training programme that involved cognitive behavioural therapy-based interventions
Jordans et al. (2010)^32^	(i) A gender-balanced group of interventionists was selected based on previous experience and affinity to work with children – they were selected from the targeted communities; and (ii) the number was not specified	(i) 15-day cognitive behavioural therapy-based skills-oriented training; and (ii) regular supervision by an experienced counsellor
Patel et al. (2010)^a^^43^	(i) 24 lay health counsellors were locally recruited and had no previous health-care background; (ii) they performed case management duties and delivered all non-drug treatments; (iii) 12 counsellors were assigned to public health-care facilities and 12 to private health-care facilities; and (iv) each health-care facility had one lay counsellor	(i) 2 months of training in an interpersonal therapy intervention; and (ii) support by a psychiatrist during the trial
Yeomans et al. (2010)^41^	(i) All of the workshops were led by Burundian lay counsellors chosen by the nonprofit organization for their extensive experience with trauma workshop facilitation and for having demographics comparable to the participants: rural, poor, many without substantial formal education, and balanced in gender and ethnicity; and (ii) a study author stated in email correspondence that the facilitators were “simply lay people in the community working as lay facilitators of workshops, either employed or not employed.”	All lay counsellors had a full day of training dedicated to the modification of the standard workshop (in which they had been previously trained)
Connolly & Sakai (2011)^30^	Twenty-eight adult women and one man from the community chosen by the community leader of a volunteer Protestant religious group	(i) 2 full days of thought field therapy training; and (ii) supervision by study authors during interventions
Ertl et al. (2011)^31^	Fourteen adults (seven women and seven men) who were community-based lay therapists without a mental health or medical background	Intensively trained local lay counsellors underwent narrative exposure therapy training for an unspecified length of time
Tol et al. (2012)^39^	(i) An unspecified number of lay counsellors, or interventionists, who had to be older than 17 years and have at least a high school education, were selected from local target communities based on a selection procedure assessing social skills through role plays; (ii) they were generally people with no formal mental health training but had some experience as volunteers in humanitarian programmes; and (iii) a study author stated in email correspondence that the interventionists “were newly hired community members, not currently employed as community health workers, etc.”	Counsellors were trained in a cognitive behavioural therapy-based intervention and supervised in implementing the intervention for 1 year before the study
Connolly et al. (2013)^29^	Thirty-six adult men and women from the community chosen by a Catholic priest or community leader on the basis of their subjective level of respect in the community	(i) 2 full days of thought field therapy training; and (ii) supervision by study authors during interventions
Meffert et al. (2014)^33^	Five members of the Sudanese community without prior mental health training were trained to deliver interpersonal therapy	(i) 1 week of training in an interpersonal therapy intervention; and (ii) group supervision once a week
O'Callaghan et al. (2014)^36^	(i) “Three male and three female local lay facilitators (total of six) living in Dungu and working for SAIPED, a Dungu-based humanitarian NGO, delivered the intervention”; and (ii) although SAIPED was referred to as an NGO, a study author stated in email correspondence that the facilitators in the Dungu pilot psychosocial study involved workers (volunteers) in a local community-based organization who had no previous mental health training	3 hours of training in a family-focused psychosocial support intervention in each of eight modules (24 hours total)
Tol et al. (2014)^38^	(i) Lay counsellors comprised an unspecified number of locally identified non-specialized facilitators trained and supervised in implementing the intervention for 1 year before the study. Facilitators had at least a high school diploma and were selected for their affinity and capacity to work with children as demonstrated in role plays and interviews; and (ii) a study author stated in email correspondence that the lay counsellors “were newly hired community members, not currently employed as community health workers, etc.”	Counsellors were trained in a cognitive behavioural therapy-based intervention and supervised in implementing the intervention for 1 year before the study
Murray et al. (2015)^34^	Twenty-three adult counsellors (11 from study sites and 12 external); their backgrounds varied but all counsellors had at least a high school education and basic communication skills	(i) 10 days of trauma-focused cognitive behavioural therapy training; and (ii) subsequent weekly meetings with supervisors and meetings with trauma-focused cognitive behavioural therapy experts
Nadkarni et al. (2015)^45^	(i) At the end of the internship, 12 lay counsellors (10 female), who achieved competence as assessed by standardized role plays, were selected for the pilot randomized control trial; and (ii) on average, lay counsellors were 25.9 years of age with 15 years of education	3 weeks of training by professional therapists in counselling for alcohol problems, a psychosocial intervention, following an internship
Robson et al. (2016)^37^	(i) The Catholic diocese selected 36 catechists who were volunteer religious education teachers or assistants to the clergy; and (ii) the catechists were well educated and respected as leaders within their communities	(i) 2 full days of training in thought field therapy; and (ii) supervision by study authors during treatments
Nadkarni et al. (2017)^46^	(i) Counsellors were adults with no prior professional training or qualification in the field of mental health, they had completed at least secondary school education, and were fluent in the vernacular language used in the study setting; (ii) 11 counsellors participated in the trial; and (iii) a study author stated in email correspondence that “The lay counsellors were not employed when we recruited them for our programme”	(i) 2 weeks of classroom training in counselling for alcohol problems, a psychosocial intervention, with a 6-month internship; and (ii) weekly peer supervision during the trial
Patel et al. (2017)^44^	(i) Eleven lay health counsellors who were members of the local community and were 18 years or older were selected to participate in the trial after an extensive training and selection process; (ii) they had completed a minimum of a high school education and did not have previous mental health training; and (iii) they were originally recruited by newspaper advertisements and through word of mouth	(i) 3 weeks of participatory training in a healthy activity programme, which involved psychosocial and psychoeducational interventions; and (ii) subsequent weekly peer-led supervision for 6 months
Dias et al. (2019)^42^	Four lay counsellors (two men and two women) were recruited via advertisements and word of mouth; all were over 30 years of age and had a bachelor’s degree in a non-health-related field and no previous training in mental health	1-week training course in depression-in-later-life therapy followed by intensive role playing

NGO: nongovernmental organization.

^a^ The study by Patel et al. in 2010 reported on two trials.^43^

**Table 2:** Study characteristics, systematic review of mental health interventions by lay counsellors in low- and middle-income countries, 2000–2019

Study	Study location	Study population	No. in intervention group	No. in control group	Primary intervention	No. of intervention sessions	Primary outcome measured	Effect size of intervention	
								Hedges’ *g*	Category^a^
Ali et al. (2003)^47^	Karachi, Pakistan (Qayoomabad community)	Adults aged 18–65 years	70	91	Cognitive behavioural therapy	8	Depression and anxiety (combined scores)	−0.608	Medium
Neuner et al. (2008)^35^	Nakivale refugee camp, Uganda	Rwandan and Somalian adults (average age: 35 years)	111	55	Narrative exposure therapy	6	PTSD	−0.549	Medium
Tol et al. (2008)^40^	Indonesia	Children (average age: 9 years)	182	211	Classroom-based cognitive behavioural therapy and creative play	15	PTSD	−0.675	Medium
Jordans et al. (2010)^32^	Nepal	Children aged 11–14 years	164	161	Classroom-based cognitive behavioural therapy and creative play	15	PTSD	−0.180	No effect
Patel et al. (2010)^b^^43^	Goa, India	Adults aged > 17 years	540	414	Interpersonal therapy	4–12	Depression	−0.327	Small
Patel et al. (2010)^b,^^43^	Goa, India	Adults aged > 17 years	387	414	Interpersonal therapy	4–12	Depression	0.160	No effect
Yeomans et al. (2010)^41^	Burundi	Adults (average age: 38.6 years)	37	38	Psychosocial education for PTSD	4	PTSD	−0.176	No effect
Connolly & Sakai (2011)^30^	Kigali, Rwanda	Adults aged > 18 years	71	74	Thought field therapy	1	PTSD	−0.781	Medium
Ertl et al. (2011)^31^	Uganda	War-exposed youth aged 12–25 years	28	28	Narrative exposure therapy	8	PTSD	−0.338	Small
Tol et al. (2012)^39^	Sri Lanka	Children in school grades 4–7 (aged 9–12 years)	199	200	Classroom-based cognitive behavioural therapy and creative play	15	PTSD	0.050	No effect
Connolly et al. (2013)^29^	Byumba, Rwanda	Adults aged > 18 years	85	79	Thought field therapy	1	PTSD	−1.351	Large
Meffert et al. (2014)^33^	Egypt	Sudanese adults in refugee camp aged 2–42 years	11	8	Interpersonal therapy	6	PTSD	−1.454	Large
O'Callaghan et al. (2014)^36^	Democratic Republic of the Congo	Children aged 7–18 years	79	80	Family-focused psychosocial support	8	PTSD	−0.405	Small
Tol et al. (2014)^38^	Burundi	Children aged 12–15 years	119	170	Classroom-based cognitive behavioural therapy and creative play	15	PTSD	0.020	No effect
Murray et al. (2015)^34^	Zambia	Children in refugee camp aged 5–18 years	131	126	Trauma-focused cognitive behavioural therapy	10–15	PTSD	−2.129	Large
Nadkarni et al. (2015)^45^	India	Male adults aged > 18 years	23	24	Counselling for alcohol problems	1–4	Alcohol use	−0.686	Medium
Robson et al. (2016)^37^	Uganda	Adults (average age: 46 years)	114	122	Thought field therapy	1	PTSD	−1.821	Large
Nadkarni et al. (2017)^46^	India	Adults aged 18–65 years	164	172	Counselling for alcohol problems	1–4	Alcohol use	−0.152	No effect
Patel et al. (2017)^44^	India	Adults aged 18–65 years	230	236	Healthy activity programme	6–8	Depression	−0.541	Medium
Dias et al. (2019)^42^	Goa, India	Adults aged > 60 years	80	84	Depression-in-later-life therapy	ND	Depression	−0.838	Large
**Total**	**NA**	**NA**	**2825**	**2787**	**NA**	**NA**	**NA**	**NA**	**NA**

NA: not applicable; ND: not determined; PTSD: post-traumatic stress disorder.

^a^ Effect sizes were categorized as: no effect (Hedges’ *g*: 0.0 to ±0.2); small effect (Hedges’ *g*: > ±0.2 to < ±0.5); medium effect (Hedges’ g: > ±0.5 to < ±0.8); and large effect (Hedges’ g: ≥ ±0.8).

^b^ The study by Patel et al. in 2010 reported on two trials.^43^

### Meta-analysis

**Fig. 2:**
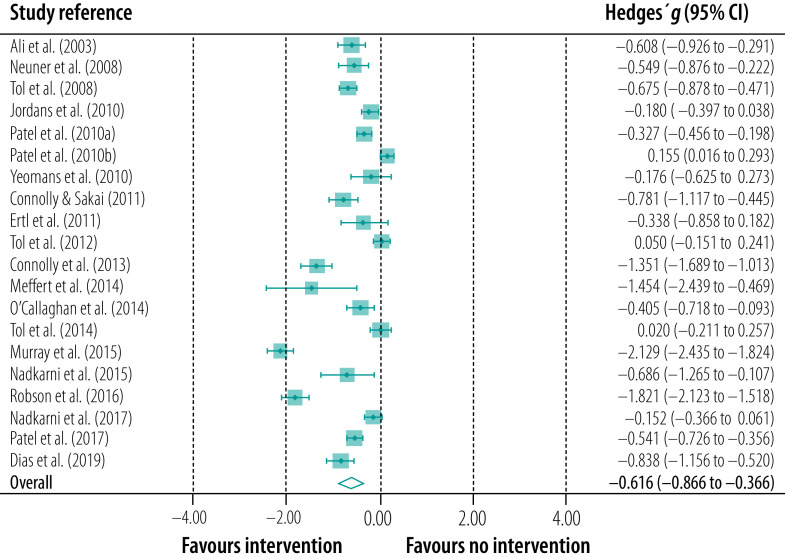
Forest plot of effect of interventions, systematic review of mental health interventions by lay counsellors in low- and middle-income countries, 2000–2019

Of the 20 trials, 14 found that the intervention by professionally trained, lay counsellors had a significant effect: in five it was a large effect, in six a medium effect and in three a small effect (Table 2 and Fig. 2). Only six trials found no significant effect. Overall, the interventions had a medium effect (i.e. Hedges’ *g*: −0.616; 95% confidence interval: −0.866 to −0.366) as calculated using the random-effects model (Fig. 2). Although the aggregate effect size calculated using the fixed-effects model was smaller (Hedges’ *g*: −0.407; 95% confidence interval: −0.460 to −0.353), it was still significant. Nevertheless, given the initial rationale, use of the random-effects model was warranted. Moreover, when the distribution of the effect sizes observed in the 20 trials was examined using the *Q* test to identify outliers, it was found that some differences were unlikely to be due to sampling variation (i.e. there was heterogeneity). This finding provides further support for using the random-effects model, which assumes that the trials are interchangeable and that not all trials drew participants from the same population.

CI: confidence interval.

Notes: The effect size of the intervention was expressed in terms of Hedges’ *g*, where (i) a Hedges’ *g* between 0.0 and ±0.2 indicates no effect; (ii) from > ±0.2 to < ±0.5 indicates a small effect; (iii) from > ±0.5 to < 0.8 indicates a medium effect; and (iv) ≥ ±0.8 indicates a large effect. The 2010 study by Patel et al. reported on two trials.^43^

We found that the data in Murray et al.’s study were not normally distributed (*P*  <  0.001).^34^ In addition, data values from Robson et al.’s study appeared to be extreme.^37^ Consequently, we performed an additional outlier analysis, which showed that the values from Murray et al.’s study were within acceptable boundaries. Moreover, this study examined post-traumatic stress disorder, which was consistent with most other studies in the meta-analysis, and it involved a substantial number of participants: 131 in the treatment group and 136 in the control group. An additional normality test performed for Robson et al.’s study failed to show a significant result. Consequently, following commonly accepted practice,^59^ we decided not to remove any studies in conducting our meta-analyses, as there were no extreme outliers.

Details of our assessment of the risk of bias in the 19 studies across all seven bias domains are available from the data repository.^24^ In summary, we found that: (i) the risk of bias due to incomplete outcome data reporting was low in 95% (18/19) of studies; (ii) the risk of bias due to random sequence generation was low in 79% (15/19); (iii) the risk of selective reporting bias was low in 68% (13/19); (iv) the risk of other sources of bias was low in 63% (12/19); (v) the risk of bias due to outcome assessment was low in 58% (11/19); (vi) the risk of allocation concealment selection bias was low in 53% (10/19); and (vii) the risk of performance bias associated with blinding participants and personnel was low in 42% (8/19).

We also assessed the 19 studies for publication bias by creating a funnel plot of Hedges’ *g* against the standard error (data repository).^24^ There was no clear pattern and there was no general shift in effect sizes to either side of the estimated aggregate mean. Although the individual study effect sizes did not appear to be randomly distributed, we determined using Duval and Tweedie’s trim-and-fill method that no study needed to be excluded,^60^ despite appearing to be an outlier. In addition, this analysis did not suggest that any individual value needed to be adjusted.

## Discussion

Our meta-analysis of 20 randomized controlled trials suggests that professionally trained, lay counsellors can provide effective mental health interventions in low- and middle-income countries. In particular, 14 of these trials found that outcomes improved significantly more in the intervention than the control group. Moreover, Hedges’ *g* for the aggregate effect size of the lay counsellor interventions on symptoms indicated a medium effect size, when calculated using a random-effects meta-analysis model.

Previous reviews have often focused on or included programmes that involve training professional or paraprofessional health workers (who are already overburdened) to deliver mental health services.^7^ We found no previous meta-analyses of therapy given exclusively by lay counsellors with which to compare our results.

Our findings may be important for health authorities, policy-makers and other stakeholders planning psychiatric health care. In particular, the use of lay counsellors could provide valuable, first-tier, mental health services for people in underserved communities. The heterogeneity of the interventions used in the studies we identified is both a strength and a weakness. On the one hand, the diversity in the type and length of treatment and training and in the supervision and support provided for lay counsellors makes it difficult to draw firm conclusions. On the other, this diversity is a strength as it demonstrates the reality of the treatment provided by lay therapists in low- and middle-income countries.

The use of lay counsellors for mental health interventions is only a partial solution to the gap in mental health provision in low- and middle-income countries. The treatment gap in these countries actually reflects deeper systemic global problems, such as the unequal distribution of resources and vast disparities in income.^13^ The use of lay counsellors could also be problematic. A qualitative investigation in South Africa found that, in several studies, the lack of formal supervision, standardized training and a clear definition of the lay counsellor’s role led to poor treatment fidelity.^61^ Other problems were inconsistent remuneration and health-care managers who did not appreciate the importance of counselling.^61^

Further research is needed on mental health interventions facilitated by lay counsellors in places where mental health needs outstrip professional resources. Although the interventions examined in this systematic review and meta-analysis show promise for reducing the mental health burden globally, they will need to be tested using more stringent methods.

The data we obtained from randomized controlled trials have several limitations. Some authors did not adequately report outcome data or provide a satisfactory description of the lay counsellors or their training. In addition, some studies involved few participants and appeared to lack adequate statistical power, whereas others did not adequately describe blinding or masking procedures. Also, the cost–effectiveness of using lay counsellors to provide different interventions will need to be evaluated. Finally, there are several mental health interventions that can be administered by professionally trained community members that have not yet been examined. They will need to be studied if the goal of closing the gap in mental health provision globally by harnessing community resources as agents of change is to be pursued seriously.

Although randomized controlled trials of mental health interventions by community members that require minimal professional therapist involvement are scarce, we identified 20 such trials. Together, these trials demonstrate that professionally trained, lay counsellors have a promising role to play in helping close the mental health treatment gap in low- and middle-income countries.
